# Selective advantage of implementing optimal contributions selection and timescales for the convergence of long-term genetic contributions

**DOI:** 10.1186/s12711-018-0392-z

**Published:** 2018-05-10

**Authors:** David M. Howard, Ricardo Pong-Wong, Pieter W. Knap, Valentin D. Kremer, John A. Woolliams

**Affiliations:** 1 The Roslin Institute and R(D)SVS, The University of Edinburgh, Edinburgh, UK; 2 Genus-PIC, Ratsteich 31, 24837 Schleswig, Germany; 3 Genus PLC, 100 Bluegrass Commons Blvd, Suite 2200, Hendersonville, TN 37075 USA; 4 Genus-PIC, Edinburgh, UK

## Abstract

**Background:**

Optimal contributions selection (OCS) provides animal breeders with a framework for maximising genetic gain for a predefined rate of inbreeding. Simulation studies have indicated that the source of the selective advantage of OCS is derived from breeding decisions being more closely aligned with estimates of Mendelian sampling terms ($\hat{a}$) of selection candidates, rather than estimated breeding values (EBV). This study represents the first attempt to assess the source of the selective advantage provided by OCS using a commercial pig population and by testing three hypotheses: (1) OCS places more emphasis on $\hat{a}$ compared to EBV for determining which animals were selected as parents, (2) OCS places more emphasis on $\hat{a}$ compared to EBV for determining which of those parents were selected to make a long-term genetic contribution (*r*), and (3) OCS places more emphasis on $\hat{a}$ compared to EBV for determining the magnitude of *r*. The population studied also provided an opportunity to investigate the convergence of *r* over time.

**Results:**

Selection intensity limited the number of males available for analysis, but females provided some evidence that the selective advantage derived from applying an OCS algorithm resulted from greater weighting being placed on $\hat{a}$ during the process of decision-making. Male *r* were found to converge initially at a faster rate than female *r*, with approximately 90% convergence achieved within seven generations across both sexes.

**Conclusions:**

This study of commercial data provides some support to results from theoretical and simulation studies that the source of selective advantage from OCS comes from $\hat{a}$. The implication that genomic selection (GS) improves estimation of $\hat{a}$ should allow for even greater genetic gains for a predefined rate of inbreeding, once the synergistic benefits of combining OCS and GS are realised.

**Electronic supplementary material:**

The online version of this article (10.1186/s12711-018-0392-z) contains supplementary material, which is available to authorized users.

## Background

Selection theory [1, 2] states that sustained genetic gain ($\Delta G$) is obtained from creating a covariance between the Mendelian sampling terms ($a$) and long-term genetic contributions ($r$) of selection candidates. This in turn provides the framework for an effective solution for the management of genomic diversity, due to the relationship between $r$ and rate of inbreeding ($\Delta F$). Various methods [3–9] have been proposed for managing genetic resources using this framework, which has become known as optimal contributions selection (OCS). OCS has been shown to generate greater $\Delta G$ for a predefined $\Delta F$, and to generate lower $\Delta F$ for a given $\Delta G$, compared to earlier methods that used truncation selection [10, 11].

So far, the source of the selective advantage provided by OCS has only been investigated in theoretical and simulation studies [7, 12, 13], with the conclusion that it arises from placing greater selection pressure on estimates of Mendelian sampling terms of selection candidates ($\hat{a}$) than in truncation selection. This study is the first to test these predictions in a real population by assessing the weightings placed on estimated breeding value (${\text{EBV}}$) versus $\hat{a}$, both before and during OCS. Given the findings of Avendaño et al. [12] and Grundy et al. [7], the following three hypotheses were tested: (1) OCS places more emphasis on $\hat{a}$ compared to ${\text{EBV}}$ for determining the initial selection of parents; (2) OCS places more emphasis on $\hat{a}$ compared to ${\text{EBV}}$ for determining which parents make a non-zero $r$; and (3) OCS places more emphasis on $\hat{a}$ compared to ${\text{EBV}}$ for determining the magnitude of $r$. Timescales for the convergence of $r$ of selected parents were also examined.

## Methods

### Population analysed

The commercial population analysed in this study consisted of 115,428 pigs comprising a single closed nucleus line that was bred continuously between 12th April 1997 and 16th August 2012, with pedigree information available for all individuals. The line was developed and also expanded over this period, leading up to the cohort studied here. ${\text{EBV}}$ that were calculated using a weighted index of traits were used at the time of selection, but the ${\text{EBV}}$ used for assessment in this study were only available at a single time point (16th August 2012). Individuals without a calculated ${\text{EBV}}$ on themselves or on their parents were excluded to ensure that the estimated Mendelian sampling term, $\hat{a}$, could be calculated for each individual. This left a total of 107,895 individuals (54,881 males and 53,014 females) for analysis, with the earliest individual being born on 23rd February 1999, which is denoted as time $t = 0$.

Woolliams et al. [1] defined the long-term genetic contribution, $r_{i\left( j \right)}$, of an ancestor $i$ to an individual $j$ as the proportion of the genes of $j$ that are expected to be derived by descent from ancestor $i$. Using this definition of $r_{i\left( j \right)}$, each individual $i$ had its long-term contribution ($r_{i}$) calculated as follows:

1 $$r_{i} = 1/n\mathop \sum \limits_{j} r_{i\left( j \right)} ,$$

where the sum $j$ was over all individuals born in the 2012 cohort and $n$ was the total number of animals in the cohort. Generation intervals were based on the definition of Bijma and Woolliams [2], such that the total number of generations at time $t$ was defined as $g\left( t \right) = \mathop \sum\nolimits_{i born \le t} r_{i}$, where the sum is over all individuals $i$ born from time 0 up to and including time $t$. Individuals of generation $g$ were defined as all individuals born at time $t$ for which $g \le g\left( t \right) < g + 1$. Using this definition, 10.4 generations were born between 23rd February 1999 and 16th August 2012, representing a generation interval of *L* = 1.29 years, with the base generation ($g = 0$) consisting of individuals born between 23rd February 1999 and 21st May 2000.

### Time scales for convergence of contributions

The value of $r_{i}$ for individual $i$ in a closed population will converge towards an asymptote over time [1]. To ensure the validity of the results from this study, $r_{i}$ was required to approach convergence. To test this convergence, contributions were examined as a function of time after birth for all individuals selected as parents and born during 1999. The final contributions for this 1999 cohort were those obtained from Eq. (1), i.e. their average contribution to the 2012 cohort, i.e. 13 years later. Contributions of the 1999 cohort to the cohorts born in each of the intermediate years, 2000–2011, were also calculated, denoted as $r_{i} \left( u \right)$, where $u$ denotes time after birth, with *u* = 1, 2, …, 12 years. For each intermediate year, the following linear regression model was used to determine the degree of convergence in that year:

2 $$r_{i} - \bar{r} = \beta \left[ {r_{i} \left( u \right) - \bar{r}} \right] + \varepsilon_{i} ,$$

where $\varepsilon_{i}$ is an error term and both the response and explanatory variables are adjusted by $\bar{r}$, which is the mean of the final contributions for all the individuals selected as parents and born during 1999. At convergence, $\beta \to 1$ and $E\left[ {\varepsilon_{i}^{2} } \right] \to 0$. This model was applied separately to contributions of males and females.

A similar methodology to that applied annually was used to assess the convergence of $r_{i}$ on a generational scale using all individuals selected as parents in the base generation $g$, i.e. $g = 0$. Contributions of individuals in the base generation to each intermediate generation were calculated for generations 1 through 8 as $r_{i} \left( g \right)$, where $1 \le g \le 8$, and final contributions were calculated as those made to $g = 9$, i.e. the final complete generation. This final generation consisted of individuals born between 6th December 2010 and 5th May 2012.

### Grouping of individuals according to the selection method

An OCS algorithm was introduced for selection in this population in 2002 and was based on the approach described by Newman et al. [14] and Kinghorn [15]. An iterative algorithm was applied to calculate a response surface, with the aim of achieving maximal $\Delta G$ for a given $\Delta F$. Prior to the introduction of this algorithm, selection had been by truncation with ad hoc restrictions aimed at controlling inbreeding. The full transition to this method of selection was completed by the end of 2005 and was used throughout the remainder of the period studied.

Individuals were assigned to either the Pre-OCS group, the OCS group, or neither of these groups using $g\left( t \right)$ defined above. Comparisons between and within the Pre-OCS and OCS groups were conducted to test the three hypotheses described below. A total of 17,165 individuals born between 23rd February 1999 and 7th December 2001 were assigned to the Pre-OCS group, which represented two generations with $g\left( t \right) < 2$. Since the OCS algorithm was introduced in 2002, only two generations were available prior to the change to OCS. A further 25,068 individuals, born between 23rd February 2006 and 7th October 2008, were assigned to the OCS group, also consisting of two generations, $5.6 \le g\left( t \right) < 7.6$. Analysing two generations in the OCS period maintained consistency with the Pre-OCS period in the analyses. In addition to $r_{i}$, the selection score ($x_{i}$) was determined for all individuals in the Pre-OCS and OCS groups from the recorded birth of offspring ($x_{i} = 1$, if the individual had offspring, and $x_{i} = 0$ otherwise); it is clear that $x_{i} = 0$ implies $r_{i} = 0$. Table 1 shows the numbers of individuals in the Pre-OCS and OCS groups by sex and contribution history.

**Table 1:** Number of males and females depending on selection group (Pre-OCS and OCS), selection score ($x_{i}$), and whether or not the long-term contribution in 2012 ($r_{i}$) was positive

Constraint		Pre-OCS		OCS	
$x_{i}$	$r_{i}$	Males	Females	Males	Females
–	–	8341	8824	13,001	12,067
> 0	–	177 (0.021)	1444 (0.164)	111 (0.009)	1839 (0.152)
> 0	> 0	118 (0.667)	219 (0.152)	35 (0.315)	179 (0.097)

The symbol ‘–’ indicates that no constraint was applied. The proportion of individuals remaining from the constraint in the previous row is in brackets

Estimates of Mendelian sampling terms for each individual $i$, $\hat{a}_{i}$, were calculated as the deviation of the individual’s ${\text{EBV}} \,({\text{EBV}}_{i} )$ from the average ${\text{EBV}}$ of its parents. In the analyses that follow, the ${\text{EBV}}_{i}$ were calculated as deviations from the mean EBV of contemporaries, defined by all animals born within 30 days before or after birth of that individual. This on average resulted in 1497 contemporaries. Unless stated otherwise, the remaining references to ${\text{EBV}}$ refer to this adjusted ${\text{EBV}}$. No adjustment for $\hat{a}_{i}$ was required since it has an expectation of 0 for all animals.

The average reliability of the ${\text{EBV}}$ across both sexes (${\text{R}}^{2}$) was approximately 0.16 in the pre-OCS period, increasing to 0.25 thereafter. Selection took place at off-test (~ 6 months of age), so both sexes had about the same volume of information available for estimating their initial breeding values. In 2012, the average ${\text{R}}^{2}$ across both sexes was 0.28.

Inbreeding coefficients ($F$) for individuals were obtained by subtracting 1 from the diagonal elements of the numerator relationship matrix [16]. Annual rates of inbreeding were calculated for the birth cohorts at year $t$ using two methods: (i) $\Delta F_{ped}$, calculated as:

3 $$\Delta F_{ped} = \left( {F_{t + 1} - F_{t} } \right)/\left( {1 - F_{t} } \right),$$

where $F_{t}$ is the average $F$ for all individuals in the cohort;

and (ii) $\Delta F_{r}$, calculated as:

4 $$\Delta F_{r} = {\raise0.7ex\hbox{$1$} \!\mathord{\left/ {\vphantom {1 4}}\right.\kern-0pt} \!\lower0.7ex\hbox{$4$}}\sum r_{i}^{2} ,$$

where the sum is over all individuals in the cohort and $r_{i}$ is defined by Eq. (1), regardless of convergence.

### Sources of selective advantages with OCS

Bivariate regressions were used to determine whether ${\text{EBV}}_{i}$ or $\hat{a}_{i}$ provided the source of the selective advantages before and during OCS. The source of the selective advantage was examined for: (1) which individuals were initially selected as parents, (2) which parents went on to make a non-zero $r_{i}$ to the population, and for (3) determined the magnitude of $r_{i}$.

#### Step 1: Initial selection as parents

Bivariate regression was used to determine whether ${\text{EBV}}_{i}$ or $\hat{a}_{i}$ was the principal variable that determined the initial selection of an individual as a parent, separately for the Pre-OCS and OCS ancestors. A generalised linear model with a binomial distribution and a logit link function was fitted together with a parameter for over-dispersion, assuming a beta-binomial distribution for $p_{i}$ conditional on the fixed effects. Let the probability of selection for individual $i$ be $\mu_{i} = E[x_{i} ]$, then if $s\left( . \right)$ is the logistic link function, the following model was fitted:

5 $$s^{ - 1} (\mu_{i} ) = \alpha + \beta_{{\rm EBV}} {\text{EBV}}_{i} + \beta_{{\hat{a}}} {\hat{a}}_{i} + \varepsilon_{i} ,$$

where $\alpha$ is an intercept and $\beta_{\text{EBV}}$ and $\beta_{{\hat{a}}}$ are the respective regression coefficients on ${\text{EBV}}_{i}$ and $\hat{a}_{i}$. Changes in deviance conditional on all other model terms were used to assess the relative importance of individual model terms. This analysis was conducted separately for each sex using the ‘quasi-binomial’ option in the ‘glm’ package of R, which estimates over-dispersion from the Pearson residuals.

#### Step 2: Maintenance of contributions over time for selected individuals

To examine the maintenance of contributions over time, models were fitted only to the subset of individuals that had been selected as parents, i.e. conditional on $x_{i} = 1$, because long-term contributions are always 0 for individuals that are not selected. For this subset, $r_{i}^{ + } = 1$ if $r_{i} > 0$, and 0 otherwise. The methods described for Step 1 were repeated with $r_{i}^{ + }$ instead of $x_{i}$, such that $\mu_{i} = E\left[ {r_{i}^{ + } } \right]$ in Eq. (5).

#### Step 3: Magnitude of long-term contributions over time for selected individuals

An intrinsic property of OCS is the targeting of a desired magnitude of $r_{i}$ for each individual. The source of the selective advantage for determining the magnitude of $r_{i}$ was assessed using: (1) all individuals that were initially selected as parents, i.e. $x_{i} = 1$, regardless of their long-term contributions, and (2) restricted to parents with a non-zero long-term contribution ($r_{i}^{ + } = 1$). The strength of the relationships of the magnitude of $r_{i}$ with both ${\text{EBV}}_{i}$ and $\hat{a}_{i}$ were investigated separately for each sex in the Pre-OCS group and in the OCS group using the following linear regression model:

6 $$r_{i} = \alpha + \beta_{{\rm EBV}} {\text{EBV}}_{i} + \beta_{{\hat{a}}} \hat{a}_{i} + \varepsilon_{i} .$$

Finally for the selected subgroup with $r_{i}^{ + } = 1$, contour plots of $r_{i}$ against ${\text{EBV}}$ and $\hat{a}$ were used to visualise and aid in interpreting the relationship of ${\text{EBV}}_{i}$ and $\hat{a}_{i}$ with the magnitude of $r_{i}$. Contour plots were produced using the ‘akima’ package [17] of R v2.15.

## Results

### Accumulation and convergence of contributions

A plot of the accumulation of $r_{i}$ over time is in Additional file 1: Figure S1, starting with the first individual born in the Pre-OCS group until the last selected individual, which was born on 23rd September 2011. The rate of accumulation was relatively constant over time, with a levelling off of the cumulative $r_{i}$ midway through 2011, which reflects individuals that were currently undergoing selection. The regression coefficient of accumulated $r_{i}$ on time was 2.14 × 10^−3^ per day, equating to an average generation interval of 468 days.

**Fig. 1:**
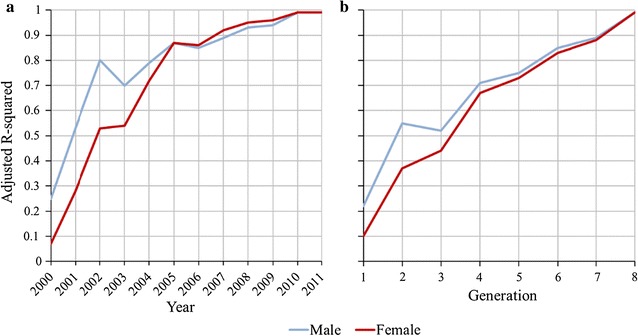
**a** Adjusted R-squared of linear regression of the final assumed long-term genetic contribution (in 2012) of all selected males and females born in 1999 ($x_{i} = 1$) on their contributions to individuals born in each year, from 2000 to 2011. **b** Adjusted R-squared of linear regression of the final assumed long-term genetic contribution (in generation 9) of all selected males and females born in generation 0 on their contributions to individuals born in each generation, from 1 to 8

Figure 1a illustrates the male and female adjusted R-squared for the model of Eq. (2) for each year. The adjusted R-squared increased rapidly at first for both males and females, with males exceeding 75% after 3 years. For males, $r$ continued to converge but at a slower rate, reaching an adjusted R-squared of approximately 95% by 9 years. Females were slower to approach convergence, with the adjusted R-squared only exceeding 75% after 5 years, but still exceeding 95% within 9 years. For selected individuals born in 1999, the standard deviation of $r_{i}$ within the 2012 cohort was 1.05 × 10^−3^.

The adjusted R-squared for the model of Eq. (2) on a generational basis is in Fig. 1b. This trend line had greater linearity than the yearly analysis. For this analysis, males again initially converged at a faster rate than females, with both sexes achieving an average adjusted R-squared of approximately 75% in five generations. For individuals in the base generation, the standard deviation of $r_{i}$ in generation 9 was 1.09 × 10^−3^.

### Rates of inbreeding over time

**Fig. 2:**
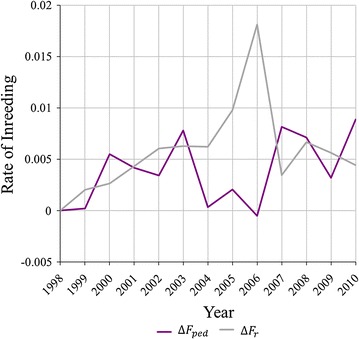
Annual rate of inbreeding based on pedigree $(\Delta F_{ped} )$ and long-term genetic contributions $(\Delta F_{r} )$)

Plots of $\Delta F_{ped}$ and $\Delta F_{r}$ over time are shown in Fig. 2. The elevated value for $\Delta F_{r}$ observed in 2006 was strongly influenced by a prolific male that sired 1419 offspring, which alone contributed 0.012 to $\Delta F_{r}$, representing 63.5% of the total contributions to $\Delta F_{r}$ for the year. The impact of this prolific individual would not be expected on a plot of $\Delta F_{ped}$, since its impact is spread across future generations beyond the endpoint in Fig. 2. $\Delta F_{r}$ is prospective in the sense that it measures the impact further down the pedigree arising from an individual’s gene flow [18, 19], whereas $\Delta F_{ped}$ is retrospective in the sense that it measures the accumulated impact of changes in contributions across all previous generations.

### Sources of selective advantages with optimal contributions selection

#### Step 1: Initial selection as parents

Estimates of regression coefficients from the logistic regression of the selection score $x_{i}$ on ${\text{EBV}}_{i}$ and $\hat{a}_{i}$ are in Table 2. There was no evidence of extra-binomial variation among Pre-OCS females (*P* ≥ 0.05) but there was significant evidence in the remaining subgroups (*P* < 0.001): the estimated parameters for over dispersion were 1.02, 1.08, 3.7 and 2.3 for Pre-OCS and OCS females, and Pre-OCS and OCS males, respectively. In the Pre-OCS group, $\hat{a}_{i}$ was negatively associated with selection in males (*P* < 0.001), but was positively associated with selection in females (*P* < 0.001). In both male and female OCS groups, both ${\text{EBV}}_{i}$ and $\hat{a}_{i}$ were significant factors (*P* < 0.001) in promoting selection as a parent, with more positive values of either favouring selection, but with $\hat{a}_{i}$ having greater regression coefficient estimates. The relative importance of these two terms differed slightly between male and female candidates. Estimates from univariate logistic regression are in Additional file 2: Table S1.

**Table 2:** Estimates of regression coefficients ($\beta$) from the bivariate logistic regression of selection score on estimated breeding values (${\text{EBV}}$) and estimated Mendelian sampling terms ($\hat{a}$)

	Pre-OCS		OCS	
	$\beta$ (s.e.)	$F$	$\beta$ (s.e.)	$F$
*Males*				
${\text{EBV}}$	− 0.12 (0.11)	1.09	0.34 (0.10)	11.88
$\hat{a}$	− 1.24 (0.33)	13.61	0.68 (0.22)	9.43
*Females*				
${\text{EBV}}$	0.01 (0.02)	0.16	0.16 (0.02)	82.24
$\hat{a}$	0.31 (0.08)	15.11	0.44 (0.04)	105.26

Standard errors (s.e.) are between parentheses. Approximate F-values are shown with numerator d.f. = 1 and denominator d.f. = 8338, 8821, 12,998, and 12,064 for Pre-OCS males and females, and OCS males and females, respectively

### Step 2: Maintenance of contributions over time for selected individuals $(x_{i} = 1)$

Estimates of regression coefficients from the logistic regression of $r_{i}^{ + }$ on ${\text{EBV}}_{i}$ and on $\hat{a}_{i}$ in the OCS period are in Table 3. In the Pre-OCS group, there was no significant effect of ${\text{EBV}}_{i}$ for either sex but a positive association with $\hat{a}_{i}$ was observed for the females (*P* < 0.05). In the OCS males, ${\text{EBV}}_{i}$ was positively associated with maintaining $r_{i}^{ + } = 1$ (*P* < 0.001), whereas $\hat{a}_{i}$ was negatively associated with maintaining a positive contribution across generations $r_{i}^{ + } = 1$ (*P* < 0.05), conditional on initial selection as a parent. Among OCS females, both ${\text{EBV}}_{i}$ (*P* < 0.001) and $\hat{a}_{i}$ (*P* < 0.05) were positively associated with maintaining a positive contribution. Estimates of univariate regression coefficients are in Additional file 2: Table S2 and show that, in these models, ${\text{EBV}}_{i}$ and $\hat{a}_{i}$ were always positively correlated with maintaining non-zero contributions. There was no evidence of extra-binomial variation (*P* ≥ 0.05) for any of the four sub-groups.

**Table 3:** Estimates of regression coefficients ($\beta$) from the bivariate logistic regression of maintenance of non-zero contributions on estimated breeding values (${\text{EBV}}$) and estimated Mendelian sampling terms ($\hat{a}$)

	Pre-OCS		OCS	
	$\beta$ (s.e.)	$F$	$\beta$ (s.e.)	$F$
*Males*				
${\text{EBV}}$	0.03 (0.12)	0.07	0.96 (0.23)	17.81
$\hat{a}$	0.10 (0.22)	0.22	− 0.65 (0.26)	6.45
*Females*				
${\text{EBV}}$	− 0.01 (0.06)	0.04	0.25 (0.06)	21.31
$\hat{a}$	0.24 (0.11)	4.37	0.22 (0.10)	5.07

Standard errors (s.e.) are between parentheses. Approximate $F$-values are also shown: with numerator d.f. = 1 and denominator d.f. = 174, 1441, 108 and 1836 for Pre-OCS males and females, and OCS males and females, respectively

#### Step 3: Magnitude of long-term contributions over time for selected individuals $(x_{i} = 1)$

Estimates of regression coefficients from the bivariate regression of $r_{i}$ on ${\text{EBV}}_{i}$ and $\hat{a}_{i}$ conditional only on $x_{i} = 1$ are in Table 4 and those conditional on $r_{i}^{ + } = 1$ are in Table 5. The additional restriction imposed by $r_{i}^{ + } = 1$ substantially increased the standard errors of the estimates and, as a result, estimates in Table 5 do not differ qualitatively from those in Table 4 if these uncertainties are taken into consideration. In males (conditional on $x_{i} = 1$), ${\text{EBV}}_{i}$ had a significant effect on $r_{i}$ during both Pre-OCS (*P* < 0.05) and OCS (*P* < 0.01). In females (conditional on $x_{i} = 1$), neither $\hat{a}_{i}$ nor ${\text{EBV}}_{i}$ had a significant effect on $r_{i}$ Pre-OCS, but in the female OCS group, $\hat{a}_{i}$ was a significant determinant of $r_{i}$ (*P* < 0.001). Estimates of univariate regression coefficients, conditional only on $x_{i} = 1$ and conditional on $r_{i}^{ + } = 1$ are in Additional file 2: Tables S3 and S4, respectively.

**Table 4:** Estimates of regression coefficients ($\beta$) from the bivariate regression of the long-term contributions ($r$) on estimated breeding values (${\text{EBV}}$) and estimated Mendelian sampling terms ($\hat{a}$) for all selected individuals

	Pre-OCS		OCS	
	$\beta$ (s.e.)	$F$	$\beta$ (s.e.)	$F$
*Males*				
${\text{EBV}}$	10.98 (5.24)	4.38	45.34 (14.37)	9.95
$\hat{a}$	− 6.97 (9.13)	0.58	9.78 (21.45)	0.21
*Females*				
${\text{EBV}}$	0.65 (0.60)	1.18	0.87 (0.51)	2.91
$\hat{a}$	0.77 (1.25)	0.39	3.21 (0.93)	11.92

Standard errors (s.e.) are between parentheses. The importance of the terms is assessed using F-values; numerator d.f. = 1, and denominator d.f. are 174, 1441, 108 and 1836 for Pre-OCS males and females, and OCS males and females, respectively

**Table 5:** Estimates of regression coefficients ($\beta$) from the bivariate regression of the long-term contributions ($r$) on estimated breeding values (${\text{EBV}}$) and estimated Mendelian sampling terms ($\hat{a}$) for all individuals with $r_{i}^{ + } = 1$

	Pre-OCS		OCS	
	$\beta$ (s.e.)	$F$	$\beta$ (s.e.)	$F$
*Males*				
${\text{EBV}}$	15.87 (7.19)	4.87	38.19 (53.82)	0.50
$\hat{a}$	− 13.92 (12.36)	1.27	53.63 (62.43)	0.74
*Females*				
${\text{EBV}}$	4.36 (3.07)	2.02	− 2.97 (5.00)	0.35
$\hat{a}$	− 4.08 (5.31)	0.59	15.97 (7.46)	4.58

Standard errors (s.e.) are between parentheses. The importance of the terms is assessed using F-values; the numerator d.f. = 1, and denominator d.f. are 115, 216, 32 and 176 for Pre-OCS males and females, and OCS males and females, respectively

**Fig. 3:**
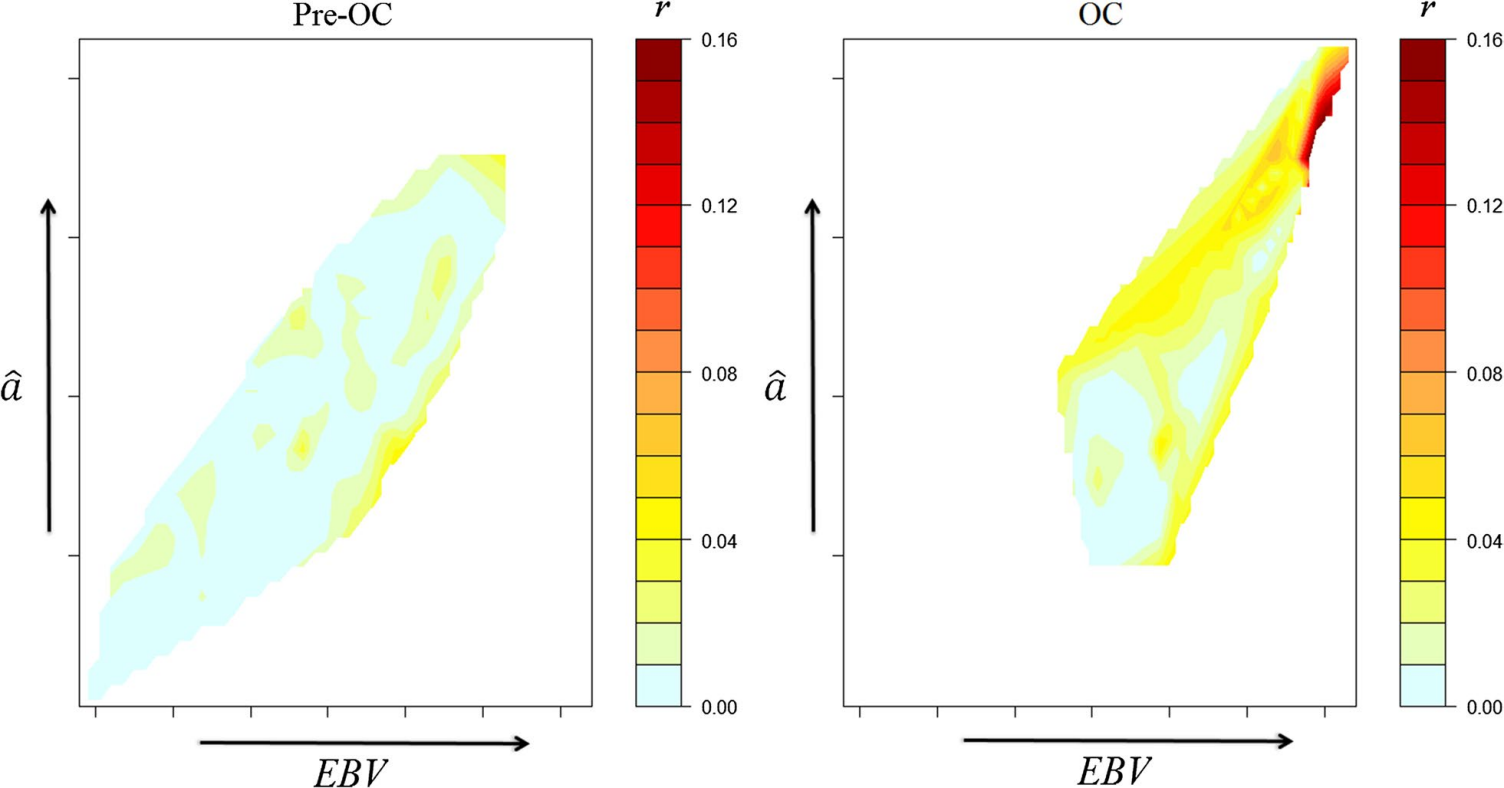
Contour plot of the magnitude of long-term genetic contributions ($r$), represented by warmth of colour, with regards to estimated Mendelian sampling terms ($\hat{a}$) and estimated breeding values (${\text{EBV}}$) for males, conditional on $r_{i}^{ + } = 1$. ${\text{EBV}}$ is plotted along the x-axis and $\hat{a}$ is plotted along the y-axis with colour gradients used to indicate the magnitude of $r$. Due to differences in the maximum value of $r$ between the sexes, a different scaling of $r$ was used between the male and female plots

**Fig. 4:**
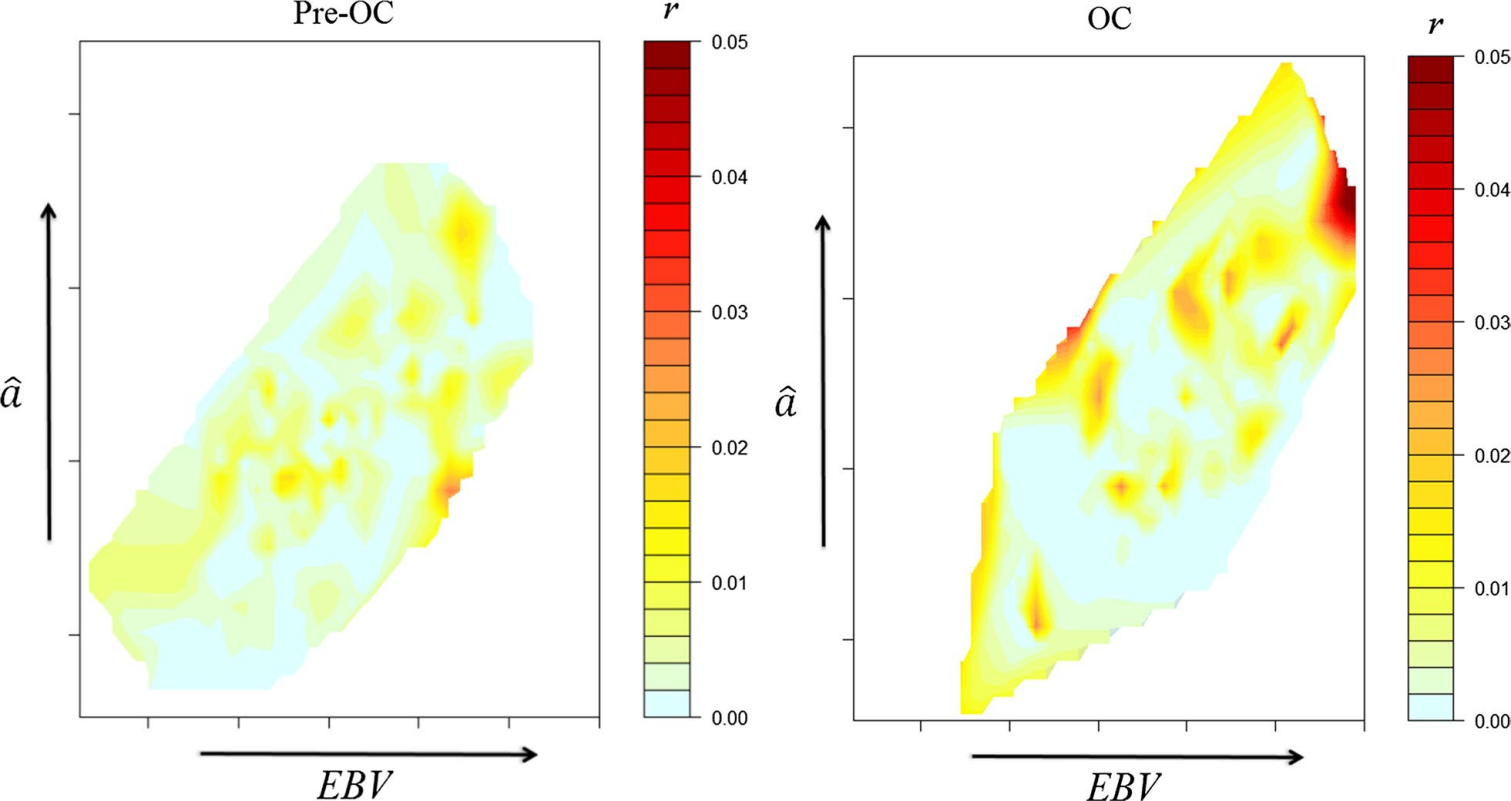
Contour plot of the magnitude of long-term genetic contributions ($r$), represented by warmth of colour, with regards to estimated Mendelian sampling terms ($\hat{a}$) and estimated breeding values (${\text{EBV}}$) for females, conditional on $r_{i}^{ + } = 1$. ${\text{EBV}}$ is plotted along the x-axis and $\hat{a}$ is plotted along the y-axis with colour gradients used to indicate the magnitude of $r$. Due to differences in the maximum value of $r$ between the sexes, a different scaling of $r$ was used between the male and female plots

A contour map illustrating the relationship of $r_{i}$ with ${\text{EBV}}_{i}$ and with $\hat{a}_{i}$ is in Fig. 3 for selected males with $r_{i}^{ + } = 1$ and in Fig. 4 for selected females with $r_{i}^{ + } = 1$. More active control of $r_{i}$ was generally observed in the OCS selection period compared to the Pre-OCS period. There was a shift in the coloured area of the plot both horizontally to the right and vertically upwards following the introduction of OCS, which shows that animals with $r_{i}^{ + } = 1$ had higher ${\text{EBV}}_{i}$ and $\hat{a}_{i}$ in the OCS period than animals with $r_{i}^{ + } = 1$ in the Pre-OCS period. Examining the colour dispersal of the Pre-OCS contour plot revealed that ${\text{EBV}}_{i}$, along the x-axis, was the more important determinant for the magnitude of $r_{i}$ in the Pre-OCS period. In the OCS period, there was a change in the distribution of $r_{i}$, with warmer colours found predominantly in the upper-most half of the contour plot. This demonstrates that after the introduction of OCS, the $\hat{a}_{i}$, on the y-axis, was more important for determining the magnitude of $r_{i}$. For males, the largest concentration of warmer colour in the OCS plot was in the upper right hand corner of the plot (Fig. 3), highlighting that both ${\text{EBV}}_{i}$ and $\hat{a}_{i}$ were important for males with the very highest $r_{i}$.

## Discussion

The research reported here was made possible by the availability of data from a single, large (n = 107,895) nucleus breeding line from Genus-PIC. This was a novel opportunity to use hypothesis-driven analyses to assess the source of the selective advantage under different selection strategies using longitudinal data. The particular hypotheses investigated were that adoption of OCS, to maximise gain conditional on the target rate of inbreeding, shifts the emphasis of selective advantages from ${\text{EBV}}$ towards estimates of Mendelian sampling terms, $\hat{a}_{i}$, based on both theory and previously published simulations [10]. The outcome of the analyses provides some qualified support for these hypotheses in females.

To maximise accuracy and minimise bias in an assessment of OCS, a number of conditions are required: (i) adequate periods of convergence for each method of selection; (ii) ${\text{EBV}}$ that are relevant to decision-making; (iii) stability in breeding objectives, selection pressures and rates of inbreeding; and, (iv) adequate population size over time. Not all these conditions were met in this commercial operation. In relation to (i), analysis of convergence of contributions for selected individuals (Fig. 1) suggested that eight generations were required to reach convergence, rather than the five generations found in simulations [1], and this was achieved only for the Pre-OCS group. However, the process of convergence within the OCS group was well advanced after five generations (Fig. 2) and this is supported by the evidence from the Pre-OCS group where the correlation between contributions at 5 and 8 generations after birth was 0.86. In relation to (ii), an individual’s ${\text{EBV}}_{i}$ and $\hat{a}_{i}$ are dynamic and increase in accuracy as more information becomes available from descendants. Selection decisions were made using ${\text{EBV}}_{i}$ and $\hat{a}_{i}$ that were available at the time of selection, but for the current study these values were only available at a single time point (16th August 2012). Therefore, the accuracies of ${\text{EBV}}_{i}$ and $\hat{a}_{i}$ used in the analyses varied among individuals. Differences in accuracy are greatest between animals selected as parents, i.e. those that have offspring, and those that were not selected. Therefore, analyses conditional on initial selection as a parent (steps 2 and 3) are likely to be less affected by variation in accuracies of ${\text{EBV}}_{i}$ and $\hat{a}_{i}$ than analyses for step 1. In relation to (iii), it is expected that changes in breeding objectives occur over time within a commercial population, and the approximate correlation between the selection index applied in 2000 and that applied in 2005 (calculated from the (co)variance matrix of the ${\text{EBV}}$ in the indices and the two sets of weights applied) was only moderate at 0.39. It is clear from Table 1 that selection intensity increased after initial establishment and expansion of the line, which affects quantitative comparisons of Pre-OCS and OCS groups. In relation to (iv), empirically, this population of more than 100,000 individuals born over the course of 15 years was sufficient to detect sources of selective advantages. The standard errors of the partial regression coefficients for ${\text{EBV}}_{i}$ and $\hat{a}_{i}$ for selected males were relatively large compared to those for females, as a result of the higher selection intensity applied to males. This higher selection intensity in males increased the magnitude of the potential effects [1], but also greatly reduced the sample size.

One advantage of the study is that it was conducted on data collected on a real population rather than simulation, but this also means that day-to-day decision-making is inevitably driven by commercial requirements rather than strictly following selection recommendations. In summary, although the Pre-OCS group acted as a practical control for the OCS-group, the commercial setting of the data indicates that comparisons between the Pre-OCS and OCS groups should be considered qualitatively and with care. However, the comparison of outcomes from the OCS group with the theoretical expectations remains valid.

### Accumulation and convergence of contributions

The observed linear increase in the accumulation of $r_{i}$ demonstrated a relatively uniform generation interval over time of approximately 468 days. Alternative generation intervals can be calculated for this population by either averaging the age of parents at birth of their first offspring (368 days), or after the birth of the first offspring that is selected (415 days). The latter is closer in concept to the generation interval in quantitative genetics since it includes the concept of replacement, and moves towards that provided by $r_{i}$, which is the length of time to renew the gene pool that is destined to maintain the population in the long term.

There was evidence that the generation interval calculated from parent ages increased over time (results not shown) and this was likely associated with operational decisions concerned with expansion of the line (see Table 1). The stochastic simulation work of Bijma and Woolliams [2] and Woolliams et al. [1] found that the generation interval calculated using $r_{i}$ was shorter than that using the average age of parents of a cohort under simple mass selection with a pre-determined age structure. The primary reason for this qualitative difference between these two methods for calculating generation intervals is that there is a selective advantage to offspring born to younger parents in the mass selection scenario, since their parents have higher breeding values than the population average. With mixed-model evaluations, e.g. best linear unbiased prediction (BLUP), the genetic trend across cohorts is accounted for, which results in more effective selection among candidates of different ages.

### Sources of selective advantages of optimal contributions selection

The OCS algorithm guides the optimum $r_{i}$ over generations until convergence, through consideration of both an individual’s ${\text{EBV}}_{i}$ as well as its degree of relatedness with the rest of the population. This approach is expected to reduce the $r_{i}$ of individuals from over-represented families, allowing the selection of superior animals from families with lower ${\text{EBV}}$. As a result, individuals with greater $\hat{a}_{i}$ are selected to provide comparatively larger $r_{i}$ [7, 20]. This process results in the maximum gain ($\Delta G$) conditional on the target rate of inbreeding ($\Delta F$), since $\Delta G \propto \varSigma r_{i} a_{i}$ (i.e. not $\varSigma r_{i} {\text{EBV}}_{i}$) and $\Delta F \propto \varSigma r_{i}^{2}$ (see Woolliams et al. [1]). In the case of perfect accuracy of ${\text{EBV}}$ (e.g. with a heritability $h^{2} = 1$) and no interdependence of generations, this would take the form of a linear allocation above a cut-off, whereby selected individuals would be those with $a_{i}$ above a threshold and $r_{i}$ is expected to be linearly related to $a_{i}$, conditional on selection [12]. Where these conditions do not hold, the relationship between $r_{i}$ and $a_{i}$ will have additional random noise and the true breeding value or ${\text{EBV}}_{i}$ will also influence $r_{i}$. To examine these different aspects, the selection process was broken down into three consecutive steps, each more closely focused on the ultimate genetic contributors to the population, and each with its associated hypothesis.

The first hypothesis was concerned with whether ${\text{EBV}}_{i}$ or $\hat{a}_{i}$ was the principal factor that determined the selection of an individual to become a parent. In practice, the selection of an individual to become a parent, unlike the development of $r_{i}$, is only influenced by selection decisions within a narrow time-period. For females, $\hat{a}_{i}$ was observed to be the most important determinant for selection of an individual to become a parent, although it was unclear if this had emerged as a result of the introduction of OCS, in spite of the influence being stronger in the OCS group.

The most convincing evidence for the importance of $\hat{a}_{i}$ in determining $r_{i}$ when using OCS selection came from analyses that addressed the third hypothesis, which related the magnitude of $r_{i}$ to ${\text{EBV}}_{i}$ and $\hat{a}_{i}$, conditional on $i$ being selected initially. Of the analyses conducted in this study, these analyses most closely followed a previous simulation study [10] and directly examine the theoretical expectation that OCS will guide contributions in direct relation to $\hat{a}_{i}$ among animals that are initially selected. The footprint of OCS that was expected from the simulation study was observed among females of the OCS group, with $\hat{a}_{i}$ positively associated with $r_{i}$ and dominating the influence of ${\text{EBV}}_{i}$. However, the reverse was observed for males, for which inferences are made more difficult due to the large standard errors of the bivariate coefficients. It is possible that short-term commercial needs that do not follow the outcome of the OCS algorithm are more likely to occur among males, compared to females, as male selection opportunities are greater and offer a more rapid response to the need.

## Conclusions

This work represents the first evaluation in commercial practice of the impact and validation of the theory underlying OCS, which predicts a closer alignment of $r_{i}$ with $\hat{a}_{i}$ than with ${\text{EBV}}_{i}$. There was some evidence of a re-weighting in the emphasis of selection away from the ${\text{EBV}}_{i}$ towards $\hat{a}_{i}$ in the final magnitude of $r_{i}$, with females providing stronger evidence for these conclusions. The evidence from males was equivocal, in part because of higher selection intensity among males, leading to smaller numbers of males with which to conduct the regression analyses. As a result, this study provides some support to the assertion that there is an advantage in combining OCS with genomic information, which can achieve more accurate prediction of $\hat{a}_{i}$ at an earlier age, to generate greater genetic progress over time, without negatively impacting the rate of inbreeding.

## Additional files

**Additional file 1: Figure S1.** Accumulation of long-term genetic contributions over time with a line of best fit.

**Additional file 2: Tables S1, S2, S3, S4.** Estimates of regression coefficients ($\beta$) from univariate regressions of selection score (*x*_*i*_) on estimated breeding values (${\text{EBV}}$) and estimated Mendelian sampling terms ($\hat{a}$). Standard errors (s.e.) are given in parentheses. **Table S2.** Estimates of regression coefficients ($\beta$) from univariate regressions of $r_{i}^{ + }$ (conditional on individual $i$ was selected) on estimated breeding values (${\text{EBV}}$) and estimated Mendelian sampling terms ($\hat{a}$). Standard errors (s.e.) are given in parentheses. **Table S3.** Estimates of regression coefficients ($\beta$) from simple linear regression of long-term genetic contribution (with all selected individuals, i.e. conditional on $x_{i}$ = 1) on estimated breeding values (${\text{EBV}}$) and estimated Mendelian sampling terms ($\hat{a}$). Standard errors (s.e.) are given in parentheses. **Table S4.** Estimates of regression coefficients ($\beta$) from simple linear regression of long-term genetic contribution (restricted to those individuals with a non-zero long-term genetic contribution) on estimated breeding values (${\text{EBV}}$) and estimated Mendelian sampling terms ($\hat{a}$). Standard errors (s.e.) are given in parentheses.
