# Supplementary material for: Selective advantage of implementing optimal contributions selection and timescales for the convergence of long-term genetic contributions
Source: Genet Sel Evol. 2018 May 10;50:24. doi: 10.1186/s12711-018-0392-z (PMC5946451; doi:10.1186/s12711-018-0392-z)
Supplement: Supplementary file 1 — Additional file 1: Figure S1. Accumulation of long-term genetic contributions over time with a line of best fit. [file 12711_2018_392_MOESM1_ESM.docx]

**Selective advantage of implementing optimal contributions selection and timescales for the convergence of long-term genetic contributions**

**Additional File 1**


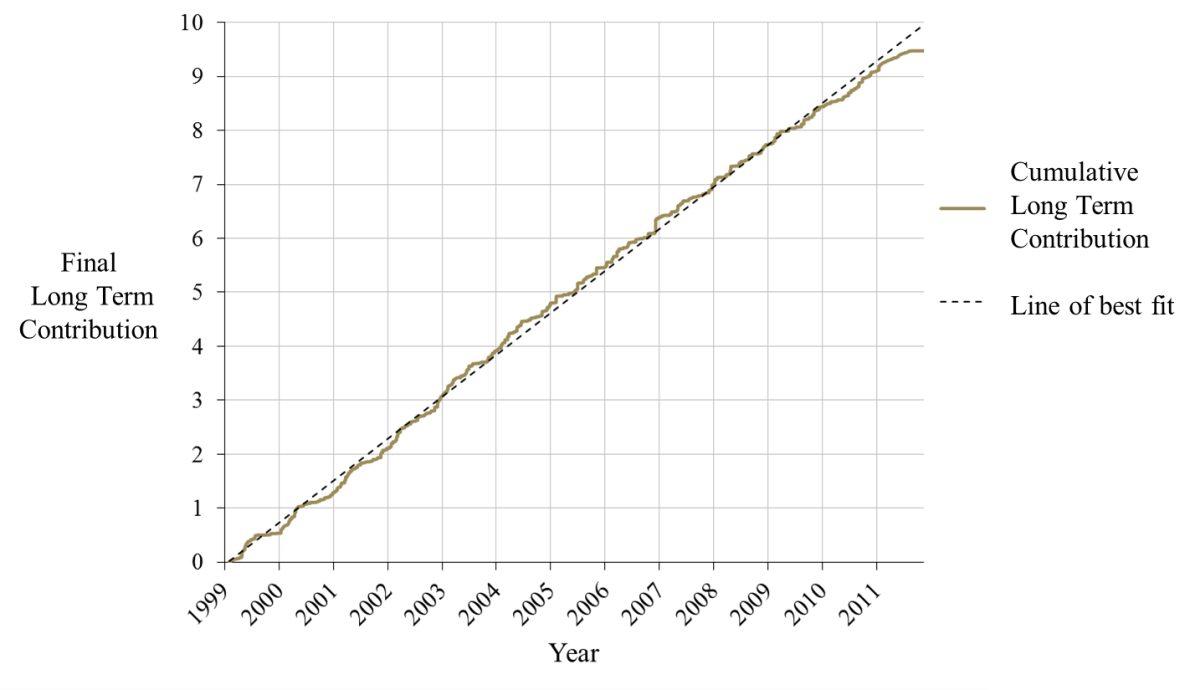


**Figure S1. The accumulation of long-term genetic contributions over time with a line of best fit**
