# Supplementary material for: Selective advantage of implementing optimal contributions selection and timescales for the convergence of long-term genetic contributions
Source: Genet Sel Evol. 2018 May 10;50:24. doi: 10.1186/s12711-018-0392-z (PMC5946451; doi:10.1186/s12711-018-0392-z)
Supplement: Supplementary file 2 — Additional file 2: Tables S1, S2, S3, S4. Estimates of regression coefficients ($\beta$β) from univariate regressions of selection score (xi) on estimated breeding values (${\text{EBV}}$EBV) and estimated Mendelian sampling terms ($\hat{a}$a^). Standard errors (s.e.) are given in parentheses. Table S2. Estimates of regression coefficients ($\beta$β) from univariate regressions of $r_{i}^{ + }$ri+ (conditional on individual $i$i was selected) on estimated breeding values ( [file 12711_2018_392_MOESM2_ESM.docx]

**Selective advantage of implementing optimal contributions selection and timescales for the convergence of long-term genetic contributions**

**Additional File 2**

**Simple linear regressions on** $\mathbf{EBV}_{\mathbf{i}}$ **and** ${\hat{\boldsymbol{a}}}_{\boldsymbol{i}}$

*Step 1. Initial selection as parents*

Table S1 contains regression coefficients obtained when fitting the univariate generalised linear models with a logistic link function (see Eq. (5)) to the selection score. The results should be compared to Table 2 which contain the bivariate regression coefficients.

**Table S1. Estimates of regression coefficients (**$\boldsymbol{\beta}$**) from univariate regressions of selection score (*x_i_*) on estimated breeding values (**$\boldsymbol{EBV}$**) and estimated Mendelian sampling terms (**$\hat{\boldsymbol{a}}$**). Standard errors (s.e) are given in parentheses.**

|  | **Pre-OC** | **OC** |
| --- | --- | --- |
|  | ***β* (s.e)** | ***β* (s.e)** |
| Males |  |  |
| $EBV$ | -0.28 (0.06) | 0.46 (0.07) |
| $\hat{a}$ | -1.41 (0.30) | 1.05 (0.19) |
| Females |  |  |
| $EBV$ | 0.04 (0.02) | 0.24 (0.02) |
| $\hat{a}$ | 0.32 (0.08) | 0.61 (0.04) |

*Step 2. Maintenance of contributions over time for selected individuals* ${(x}_{i}$ *= 1)*

Table S2 contains regression coefficients obtained when fitting the univariate generalised linear models with a logistic link function (see Eq. (5)), to $r_{i}^{+}$ defined for all selected individuals ($x_{i}$ = 1) so that $r_{i}^{+}$ = 1 if $r_{i}$ > 0, and 0 otherwise. The results should be compared to Table 3 which contain the bivariate regression coefficients.

**Table S2. Estimates of regression coefficients (**$\boldsymbol{\beta}$**) from univariate regressions of** $r_{i}^{+}$ **(conditional on individual** $\boldsymbol{i}$ **was selected) on estimated breeding values (**$\boldsymbol{EBV}$**) and estimated Mendelian sampling terms (**$\hat{\boldsymbol{a}}$**). Standard errors (s.e) are given in parentheses.**

|  | **Pre-OC** | **OC** |
| --- | --- | --- |
|  | ***β* (s.e)** | ***β* (s.e)** |
| Males |  |  |
| $EBV$ | 0.08 (0.07) | 0.59 (0.14) |
| $\hat{a}$ | 0.15 (0.13) | 0.20 (0.15) |
| Females |  |  |
| $EBV$ | 0.06 (0.04) | 0.32 (0.05) |
| $\hat{a}$ | 0.22 (0.09) | 0.47 (0.08) |

*Step 3. Magnitude of long-term contributions over time for selected individuals* ${(x}_{i}$ *= 1)*

Table S3 contains regression coefficients obtained from simple linear regression linear to $r_{i}$ with all selected individuals, i.e. conditional on $x_{i}$ = 1. Table S4 shows the same information when the regression is restricted to only those individuals with$r_{i}^{+}=1$, so that individuals with offspring that ultimately leave no descendants are excluded. The results may be compared to Tables 4 and 5 respectively which contain the corresponding bivariate regression coefficients.

**Table S3. Estimates of regression coefficients (**$\boldsymbol{\beta}$**) from simple linear regression of long-term genetic contribution (with all selected individuals, i.e. conditional on** $\boldsymbol{x}_{\boldsymbol{i}}$ **= 1) on estimated breeding values (**$\mathbf{EBV}$**) and estimated Mendelian sampling terms (**$\hat{\boldsymbol{a}}$**). Standard errors (s.e) are given in parentheses.**

|  | **Pre-OC** | **OC** |
| --- | --- | --- |
|  | ***β* (s.e)** | ***β* (s.e)** |
| Males |  |  |
| $EBV$ | 7.72 (3.05) | 49.76 (10.59) |
| $\hat{a}$ | 8.58 (5.36) | 55.33 (16.49) |
| Females |  |  |
| $EBV$ | 0.87 (0.48) | 1.88 (0.42) |
| $\hat{a}$ | 1.58 (1.00) | 4.10 (0.77) |

**Table S4. Estimates of regression coefficients (**$\boldsymbol{\beta}$**) from simple linear regression of long-term genetic contribution (restricted to those individuals with a non-zero long-term genetic contribution) on estimated breeding values (**$\mathbf{EBV}$**) and estimated Mendelian sampling terms (**$\hat{\boldsymbol{a}}$**). Standard errors (s.e) are given in parentheses.**

|  | **Pre-OC** | **OC** |
| --- | --- | --- |
|  | ***β* (s.e)** | ***β* (s.e)** |
| Males |  |  |
| $EBV$ | 9.92 (4.11) | 76.06 (30.76) |
| $\hat{a}$ | 8.47 (7.17) | 89.91 (35.56) |
| Females |  |  |
| $EBV$ | 2.86 (2.36) | 3.95 (3.86) |
| $\hat{a}$ | 0.73 (4.10) | 13.11 (5.69) |
